# Editorial: From North to South: a global perspective on the impact of endocrine disruptors on reproductive development and function

**DOI:** 10.3389/ftox.2026.1915721

**Published:** 2026-07-14

**Authors:** Cassy M. Spiller, Andrea B. Webster, Terje Svingen, Anderson J. Martino-Andrade

**Affiliations:** 1 School of Biomedical Sciences, The University of Queensland, Brisbane, QLD, Australia; 2 Mammal Research Institute, University of Pretoria, Pretoria, South Africa; 3 National Food Institute, Technical University of Denmark, Kongens Lyngby, Denmark; 4 Departamento de Fisiologia, Universidade Federal do Paraná, Curitiba, Brazil

**Keywords:** anthropogenic chemicals, chemical regulation, endocrine disrupting chemicals (EDC), exposure, human health

Exposure to endocrine-disrupting chemicals (EDCs) is a pervasive global health concern. These anthropogenic substances can interfere with hormonal signalling pathways that govern development, growth, metabolism, and reproduction, and by so doing contribute to a wide spectrum of adverse health effects such as infertility, altered pubertal onset, and reproductive tract abnormalities (Parent et al., 2025; Skakkebaek et al., 2016). Importantly, exposure to EDCs is not geographically confined: these substances are now detectable across ecosystems worldwide, reflecting their widespread production, use, and persistence in the environment (Klingelhöfer et al., 2025; Onyime and Nworie, 2026).

While EDCs represent a shared global challenge, the scientific and regulatory responses to these chemicals are far from evenly distributed. Research activity is heavily concentrated in a limited number of high-income countries, with a pronounced North–South divide in publication output and research capacity (Klingelhöfer et al., 2025). Similarly, regulatory frameworks can vary substantially between regions, shaped by differences in socio-economic priorities, infrastructure, and policy development (Clahsen et al., 2019; Holmer et al., 2025; OECD, 2010) This imbalance has important consequences; regions with the most limited capacity for monitoring, research, and regulation are often those where exposure risks may be substantial, due to agricultural practices, industrial activities, or continued use of legacy chemicals (Bertoncello Souza et al., 2018; Clahsen et al., 2019; Gore et al., 2024; Kassotis et al., 2020; OECD, 2010) Beyond quantitative and qualitative differences in EDC exposures, variation in genetic background, nutrition, socio-economic conditions, and infectious disease burden across global regions may further shape the diverse health outcomes associated with chemical exposure (Dlamini et al., 2025; Wallis et al., 2021).

The Research Topic contributes to addressing this gap. Although comprising a modest number of articles, it highlights region-specific exposures, population contexts, and research challenges that remain underrepresented in the broader literature. In doing so, this Research Topic reiterates the need for more inclusive, globally representative research and regulation activities. It is clear that tackling the impact of EDCs on reproductive health requires coordinated international efforts, including strengthening research capacity in underrepresented regions, harmonizing regulatory approaches, and fostering global collaborations that better reflect the diversity of exposures and vulnerabilities worldwide. The contributions to this Research Topic collectively illustrate the diversity of EDC exposures and contexts across different regions and populations.

The first article (Doná et al.) examines glyphosate exposure in women from agriculturally intensive regions in Argentina and its association with breast cancer risk. Although urinary glyphosate concentrations did not differ between cases and controls, residential proximity to agricultural fields was associated with an increased risk. The study highlights both the ubiquity of glyphosate exposure in this population and the challenges of linking biomarkers of exposure to disease outcomes, underscoring the need for broader and more integrative approaches to exposure assessment.

The second article (Clark) provides a review on environmental and occupational risks faced by women in U.S. military service, an often-overlooked context of exposure. Military environments involve a complex mixture of chemical exposures that may interfere with endocrine function and reproductive health, particularly during key reproductive years. The review identifies substantial gaps and inconsistencies in the available literature, emphasizing the need for improved exposure characterization and longitudinal studies to better understand potential links between military-related exposures and adverse reproductive outcomes.

The third contribution (Vandenberg et al.) focuses on pesticides as a major class of chemicals with endocrine-disrupting and reproductive toxicant properties. It highlights the substantial global increase in pesticide use over recent decades and the implications for both occupationally exposed populations and the general population. This review highlights that pesticides remain integral to modern agriculture, but also that their widespread use raises concerns regarding unintended effects on human health, wildlife, and ecosystem integrity, placing them within the broader context of global health and planetary boundaries.

The fourth article (Passoni et al.) shifts attention to pharmaceuticals as potential endocrine disruptors, examining the use of mild analgesics during pregnancy in a Brazilian cohort. The study reports a high prevalence of analgesic use, particularly paracetamol, during early pregnancy. Given growing concerns regarding the effects of prenatal exposure to certain pharmaceuticals on reproductive and developmental outcomes (Bauer et al., 2021), these findings provide important population-level insight while reinforcing the need for further research into the potential long-term consequences of such exposures, including neurodevelopmental and reproductive effects.

Ultimately, a global perspective is essential for understanding the true health burden of chemical exposures. Recognising and addressing regional differences will be crucial for developing equitable regulatory frameworks, improving risk assessment, and ensuring that populations worldwide are adequately protected from the adverse effects of chemical exposure.
